# Association between psychological factors and temporomandibular disorders in adolescents of rural and urban zones

**DOI:** 10.1186/s12903-021-01485-4

**Published:** 2021-03-20

**Authors:** Claudia Restrepo, Ana Maria Ortiz, Ana Carolina Henao, Ruben Manrique

**Affiliations:** CES-LPH Research Group, Universidad CES, Calle 10 A No. 22-04, Medellín, Colombia

**Keywords:** Adolescents, Temporomandibular disorders, Anxiety, Depression, Somatization, Rural, Urban

## Abstract

**Background:**

Temporomandibular disorders (TMD) are related to psychological factors. Adolescence is one of the stages in life with more psychosocial vulnerability, which is dissimilar in rural and urban zones. Thus, the aim of this investigation was to evaluate the association between psychological factors (symptoms of anxiety, depression and somatization) and TMD in adolescents between 12 and 15 years, belonging to urban and rural zones of Colombia.

**Methods:**

180 subjects aged 12–15 years (mean 13.8, SD 1.2), enrolled in public schools in the rural (n = 90) and urban (n = 90) zones were included. All subjects were evaluated using the DC/TMD instrument; the Axis I was applied for the clinical examination and the Axis II for the psychological evaluation. Data were analyzed by means of T-student, Mann–Whitney, Kruskall–Wallis tests, Pearson Chi square and multiple-variable analysis with logistic regression.

**Results:**

Forty percent of the included subjects presented some type of TMD. TMD related to pain were the most common (25.5% of the studied adolescents), being myalgia the most common (20% of the adolescents in urban zone and 31.1% of the adolescents in the rural zone). There was no difference between the TMD present in males and females, but there were differences in the symptoms of Anxiety, and Somatization (p < 0.05). TMD and psychological factors were more prevalent in children with 13 years of age. A statistically significant association between TMD and symptoms of Anxiety (Pearson Chi squared 25.57, p = 0.04), depression (Pearson Chi squared 33.28, p = 0.03) and somatization (Pearson Chi squared 25.79, p = 0.04) was found in subjects from rural zones. No associations between psychological aspects and TMD were found in subjects from urban zones, but overall all psychological factors significantly influenced TMD.

**Conclusion:**

This study indicates Myalgia to be the most prevalent TMD in studied Colombian adolescents. Pain-related TMDs are associated with psychological factors in the adolescent population of rural Colombia. Symptoms of anxiety, depression and somatization were found to be associated to TMD, even when the frequency was not necessarily severe.

## Background

According to the American Academy of Orofacial Pain, TMD is defined as a collection of medical and dental conditions affecting the (TMJ) and/or the muscles of mastication, as well as contiguous tissue components [1]. They occur between 7 and 30% of adolescents [2, 3]; being diagnoses related to pain (Myalgia, Miofascial pain, Referred Myofascial Pain, Arthralgia and Headache attributed to TMD) more common than intra-articular disorders [3]. In fact, TMD pain-related frequently causes disability [4]. In Colombia, these data are not available, since the study of TMD was excluded from the National Oral Health Study (ENSAB) IV [5] and no validated tools have been used to make reliable diagnoses.

The pain caused by TMD is considered chronic [6, 7]. According to the categorization of pain included in the International Classification of Diseases (ICD)-11, to be classified as chronic, pain should affect one or more regions, be persistent and affect the daily functioning of the person [8]. Chronic pain (including those inherent in TMD) is closely related to psychological factors [9, 10], which appear to be common in adolescence [11].

According to UNICEF data [12], about 20% of teenagers worldwide have mental or behavioral problems. Depression is the disease that contributes the most to the global burden of morbidity of 15–19 years-old people [12]. Mental problems of young people who do not receive assistance, are associated with poor results in education, unemployment, drug use, violence, crimes and irresponsible sexual and reproductive activity. All these factors increase the risks of neuropsychiatric diseases [13] and mental disorders such as depression and schizophrenia [14]. The mental health problems of adolescents entail high social and economic costs because, as time passes, they can generate disabilities [12].

A high rate of Colombian adolescents (19.8%) [15], has been subjected to conditions that involve fear, affliction, violence [16–18], and educational inequality [19], among others. These situations cause this population to have a high risk of depression and somatization [12, 20], psychological conditions that are related to TMD [21]. Because social conditions are different in urban and rural zones of Colombia [17, 19], it is reasonable to think that there are differences in the association between psychological factors and TMD between both populations.

Although the mental health law in Colombia [20], protects children and adolescents as a priority, living in a society that is still considered violent for most adolescents, has consequences not only on mental health, but also on physical health. Therefore, the research question was; Is there an association between psychological factors and TMD in adolescents of Colombian rural and urban zones? To solve it, the objective of this study was to evaluate the association between psychological factors (symptoms of anxiety, depression and somatization) and TMD in adolescents between 12 and 15 years, belonging to urban and rural zones of Antioquia, Colombia.

## Methods

### Design

A cross-sectional study was conducted.

### Population and sample

Adolescents of both genders between 12 and 15 years, were included in the study. Subjects belong to the only public school with middle and high school grades of the Municipality of Fredonia (rural zone) and a public school of the Municipality of Medellín (urban zone), which was randomly chosen from the group of educational institutions attached to the Secretary of Education of Medellin, Colombia (https://www.datos.gov.co/Educaci-n/Directorio-Establecimientos-Educativos-Municipio-d/pjcr-m27u). The school was selected by an official of the Secretary of Education, which was not participating in the study.

The sample calculation was performed in Epidat, version 4.0, taking into account a population of 250 adolescents between 12 and 15 years in each educational institution to be evaluated (according to data from the Ministry of National Education of Colombia). The power was set in 90% and the margin of error in 5%. With an expected proportion of psychological factors in adolescents with TMD of 31.1 [22], the sample size needed was 150 subjects; but with an expected proportion of TMD in adolescents of 35.9 in urban area and 38.5 in rural area [22], the total sample was 180 subjects. It was decided to take the highest number of individuals required in the calculation. Thus, 90 students from Medellín, Antioquia, Colombia (urban zone) and 90 students from Fredonia, Antioquia, Colombia (rural zone) were included. Subjects were chosen at random, using the skipped list method two by two, based on the students enrolled in each of the institutions. The participants were not matched by age or gender.

Inclusion criteria were adolescents assisting to one of the institutions of the Colombian educative system. Exclusion criteria were malformations in the face or cervical spine (known by the parents or the subject), cleft lip and palate or syndromes affecting morphology or facial function. Data were collected between February and November 2018.

### Clinical evaluation of temporomandibular disorders

All subjects were evaluated using the Diagnostic Criteria for Temporomandibular Disorders (DC/TMD) [23], in its Spanish version [24], which is the reference standard for the diagnosis of TMD. In the case of the clinical examination, Axis I was used, which consists of a questionnaire of symptoms and subsequently a detailed examination of the muscles and the TMJ, at rest and function. In this examination, an evaluation is carried out on the patient, following already determined commands related to pain location, headache location, incisal relations, opening pattern, opening movement, laterality and protrusion movements, articular noises during the movement of opening and closing the mouth, joint block and muscle pain on palpation. For this last point, an algometer (LACA®121442) was used to standardize the pressure applied in each of the bands of the Temporal (anterior, middle and posterior) and Masseter (origin, middle and insertion) muscles. With the diagnostic tree, the route is followed to obtain the diagnosis or diagnoses related to pain or intra-articular disorders. The sensitivity of the instrument is ≥ 0.86 and 0.80 respectively for TMD related to pain and intra-articular disorders. The specificity is ≥ 0.98 for pain diagnoses and 0.97 for intra-articular ones. The exam was conducted within the educational institutions of Medellín and Fredonia, in an office, with the participants sitting in a school chair and with their heads on a wall. One examiner performed the clinical evaluation and another was the scorer. Both of them were previously calibrated both to perform the clinical evaluation and to fill the score sheets (Please see the error of method section).

### Psychological assessment

The psychological factors of the rural and urban regions were analyzed in advance, to guarantee there were differences between the urban and rural zones. Data were obtained from official statistics of the Colombian government, which are presented in Table 1 [25, 26]. Except for the percentage of people treated for mental and behavioral disorders, due to use of psychoactive substances, the other analyzed statistics, showed a more adverse psychological environment in the rural zone than in the urban zone. For the evaluation of psychological factors associated with TMD in the study population, Axis II of the DC/TMD [23] was used. With instruments validated for adolescents included in Axis II, the symptoms of anxiety [27, 28], depression [29, 30] and somatization [31, 32] were assessed. The Axis II instruments were applied during a school day, in the classroom of the educational institutions located in the urban and rural zones.

**Table 1:** Psychosocial aspects in the population of Fredonia (rural zone) and Medellín (urban zone). Data 2017

Zone	Incidence of domestic violence against women [22] (%)	Incidence of suicide attempt [22] (%)	Rate of students enrolled in secondary basic education [22] (%)	Percentage of people treated for mental and behavioral disorders [22] (%)	Percentage of people treated for mental disorders, including symptomatic disorders [23] (%)	Percentage of people treated for mental and behavioral disorders due to the use of psychoactive substances [23] (%)	Rate of years of life potentially lost due to emotional and behavioral disorders that usually appear in childhood and adolescence [23] (%)
Rural zone	55	13.5	64.66	3.64	0.33	0.09	21.4
Urban zone	25	7	90	0.52	0.34	0.29	2.4

### Error of method

In March 2017, calibration was performed for the use of the DC/TMD Axis I. In the calibration, a member of the International Network for Orofacial Pain and Related Disorders (INfORM) consortium participated as a reference standard, who is an official calibrator for the instrument and researcher of this study (CR). Initially, a two-day course was held for instructional and practical sessions. For the two-day session, the subsequent steps were followed: In a first session, a detailed explanation of the Axis I of the DC/TMD was made, using an instructional document and a video. Subsequently, the reference examiner (CR), performed the detailed examination to one of the researchers to be calibrated (AMO), who acted as a patient. Meanwhile, the other researcher watched (ACH) and acted as a scorer. The procedure was repeated for the other investigator. During this practice, immediate and detailed feedback was delivered. In the second session, the researchers conducted the exam with each other, with feedback from the reference investigator (CR). The third and fourth sessions included 18 patients (10 with some type of TMD and 8 controls), which were evaluated by CR and the two investigators (AMO and ACH). The data were recorded in a standardized database in Excel data were analyzed in STATA (StataCorp, College Station, TX, USA). The Kappa values obtained for the researchers were 0.85 and 0.88 respectively. For the evaluation of symptoms of depression, somatization and anxiety, already validated instruments were used, which are self-responding and do not require examiner calibration (they were mentioned in the psychological factors section of the methodology).

### Statistical analysis

All the data obtained in the exams were recorded in a digital database in Excel. In order to verify the quality of the records, 5% of the data of the subjects included in the sample were randomly assessed, in order to establish the degree of agreement between data manually recorded with Axes I and II of the DC/TMD and the data registered in the database. The data were accepted when there was a 99% or higher agreement. Frequencies were obtained for each of the variables evaluated. Operationalization and type of variables are presented in Table 2.

**Table 2:** Operationalization and type of variables included in the investigation

Variable	Operationalization	Type of variable
Age	Age in years	Quantitative continuous Interval: 12 years age group 13 years age group 14 years age group 15 years age group
Gender	Female or male gender	Qualitative Discrete dichotomous
Zone	Rural or urban	Qualitative Discrete dichotomous
TMD diagnoses	Items from each section both of the symptoms questionnaire and the clinical examination are used as part of the diagnostic algorithms for each disorder within the DC/TMD Axis I. Each diagnosis (Myalgia, Miofascial pain, Referred Myofascial Pain, Arthralgia, Headache attributed to TMD, Disc Displacement with Reduction, Disc displacement with reduction with intermittent blocking, Disc displacement without reduction with limited opening, Disc displacement without reduction without limited opening) was dichotomized as present of absent and taken as independent variable	Qualitative Discrete dichotomous
Presence of TMD	Presence of TMD was considered when at least one TMD diagnosis (Pain-related or intraarticular) was present in the sample	Qualitative Discrete dichotomous
Presence of pain-related TMD	Presence of Pain-related TMD was considered when at least one Pain-related TMD diagnosis (Myalgia, Miofascial Pain, Referred Myofascial Pain, Arthralgia or Headache attributed to TMD) was present in the sample	Qualitative Discrete dichotomous
Symptoms of anxiety	The GAD-7 is comprised of 7 items assessing anxious mood and behavior. The anxiety items are interpreted quantitatively A total sum score is computed	Quantitative continuous Interval: Categorization: Scores of 5, 10, and 15 represent cut-points for mild, moderate, and severe anxiety, respectively
Symptoms of depression	The PHQ-9 is comprised of 9 items assessing depressed modo. The depression items are interpreted quantitatively, while the life interference item is interpreted qualitatively. A total sum score is computed	Quantitative continuous Interval: Categorization: Scores of 5, 10, 15, and 20 represent cut-points for mild, moderate, moderately severe and severe depression, respectively
Symptoms of somatization	The PHQ-15 is comprised of 15 items and assesses non-specific physical symptoms, also referred to as functional symptoms or medically unexplained symptoms. A total sum score is computed	Quantitative continuous Interval: Scores of 5, 10, and 15 represent cut-points for low, medium, and high physical symptoms, respectively

Kolmogorov–Smirnov was used to test the normality of the variables age group and the psychological aspects (anxiety, depression and somatization).

TMD diagnoses and psychological factors were compared between males and females by means of Fisher and Pearson Chi-square tests and between rural and urban zones with the Pearson Chi squared test.

Differences of TMD diagnoses and psychological factors among age groups were assessed using Kruskal–Wallis and Dunn’s post-hoc tests.

Finally, presence of TMD and presence of Pain-related TMD were taken as dependent variables for two multiple-variable analyses. Zone (urban or rural) and the psychological variables (symptoms of anxiety, depression and somatization) were included as independent variables.

Data were analyzed in STATA (StataCorp, College Station, TX, USA). The results were considered significant when p value was < 0.05.

## Results

A total of 180 adolescents between 12 and 15 years (average 13.8, SD 1.2) were included in this study. In the rural zone, 90 subjects participated (41 male and 49 female; average age 14.2 years; SD 0.8). In the urban zone, the 90 subjects were 33 male and 57 female, with an average age of 13.5 years; SD 1.3). There were no losses due to lack of information in the instruments or due to withdrawals from the investigation. Forty percent of the subjects presented some type of TMD. TMD related to pain predominated (Table 3). Of the intra-articular disorders, there were only 5 cases of disc displacement with reduction.

**Table 3:** Age, gender and prevalence of Temporomandibular Disorders in the total sample, and rural and urban zone

Variable	Total sample	Urban zone	Rural zone
Age Mean (SD)	13.8 (1.2)	13.5 (1.3)	14.2 (0.8)
Gender n (%)			
Female	106 (58.8)	57 (31.6)	49 (27.2)
Male	74 (41.1)	33 (18.3)	41 (22.7)
Without TMD n (%)	108 (60)	61 (33.88)	47 (26.11)
TMD diagnoses			
Myalgia n (%)	46 (25.5)	18 (10)	28 (15.5)
Miofascial Pain n (%)	1 (0.55)	0 (0)	1 (0.55)
Referred miofascial pain n (%)	1 (0.55)	0 (0)	1 (0.55)
Arthralgia n (%)	8 (4.44)	3 (1.66)	5 (2.77)
Headache attributed to TMD n (%)	11 (6.11)	7 (3.88)	4 (2.22)
Disc displacement with reduction n (%)	5 (2.77)	1 (0.55)	4 (2.22)
Disc displacement with reduction with intermittent blocking n (%)	0	0	0
Disc displacement without reduction with limited opening n (%)	0	0	0
Disc displacement without reduction without limited opening n (%)	0	0	0

TMD, temporomandibular disorders

There was no difference between the TMD present in males and females. Regarding the psychological factors, 43.33% of adolescents having symptoms of anxiety higher than “no-minimum” were females and differences were statistically significant between genders. Somatization was higher in males than females (p = 0.001) and there were no differences between genders, when comparing depression (Table 4).

**Table 4:** Comparison of temporomandibular disorders and psychological aspects between males and females

Variable	Males n (%)	Females n (%)	p value
Myalgia	14 (7.7)	32 (17.7)	0.08*
Miofascial pain	0 (0)	1 (0.55)	
Referred miofascial pain	1 (0.55)	0 (0)	
Arthralgia	5 (2.77)	6 (3.33)	
Headache attributed to TMD	6 (3.33)	12 (6.66)	
Disc displacement with reduction	4 (2.22)	2 (1.11)	
Symptoms of anxiety	22 (12.22)	78 (43.3)	0.03**
Symptoms of depression	58 (32.22)	52 (28.8)	0.08**
Symptoms of somatization	86 (47.7)	30 (20)	0.001**

TMD, temporomandibular disorders

*Fisher test. ** Pearson Chi-square test

TMD disorders were more prevalent in children with 13 years of age, whilst psychological factors were present in all age groups (Table 5). Statistically significant differences among groups were found only for Myalgia in the case of TMD, where Post-Hoc test indicated the difference to be located between the groups with 14 and 15 years of age. In the case of psychological factors, statistically significant differences were found for symptoms of Anxiety and Depression (p < 0.05). In the case of Anxiety, the difference was located between adolescents with 12 and 14 years. In the case of Depression, the difference was between adolescents with 12 and 13 years of age (Table 6).

**Table 5:** Comparison of temporomandibular disorders and psychological aspects among age groups. Kruskal–Wallis and Dunn’s post-hoc test

Variable	Age groups				F	p value	Post-Hoc Test p-value
	12 years	13 years	14 years	15 years			
Myalgia	12 (6.66)	16 (8.88)	14 (7.77)	4 (2.22)	3.1	0.03	0.02 (14–15 years)
Miofascial pain	0	1 (0.55)	0	0	–	–	–
Referred miofascial pain	0	1 (0.55)	0	0	–	–	–
Arthralgia	0	8 (4.44)	0	0	–	–	–
Headache attributed to TMD	1 (0.55)	4 (2.22)	3 (1.66)	3 (1.66)	0.75	–	–
Disc displacement with reduction	1 (0.55)	2 (1.11)	1 (0.55)	1 (0.55)	0.33	–	–
Symptoms of anxiety	18 (10)	16 (10.66)	38 (21.11)	28 (15.55)	1.9	0.04	0.03 (12–14 years)
Symptoms of depression	23 (12.77)	42 (23.33)	18 (10)	17 (9.44)	1.8	0.03	0.03 (12–13 years)
Symptoms of somatization	27 (15.00)	34 (18.88)	29 (16.11)	26 (14.44)	1.2	0.08	–

**Table 6:** Association between psychosocial factors and temporomandibular disorders in the total sample

	Myalgia n (%)	Myofascial pain n (%)	Arthralgia n (%)	Headache attributed to TMD n (%)	Disc displacement with reduction n (%)	Fisher test	*p value*
Symptoms of anxiety							
Minimal-no anxiety	12 (26.09)	0 (0)	5 (62.50)	6 (54.55)	1 (20.00)	25.57	0.04
Mild	14 (30.43)	1 (50.00)	1 (12.50)	3 (27.27)	1 (20.00)		
Moderate	14 (30.43)	0 (0)	1 (12.50)	1(9.09)	1 (20.00)		
Severe	6 (13.04)	1 (50.00)	1 12.50)	1(9.09)	2 (40.00)		
Symptoms of depression							
Minimal-No depression	8 (17.39)	0 (0)	5 (62.50)	6 (54.55)	2 (40.00)	29.23	0.02
Mild	18 (39.13)	0 (0)	2 (25.00)	2 (18.80)	0		
Moderate	10 (21.74)	1 (50.00)	0	3 (27.27)	0		
Moderate-severe	5(10.87)	0 (0)	0	0	1 (20.00)		
Severe	5 (10.85)	1 (50.00)	1 (12.5)	0	3 (60.00)		
Symptoms of somatization							
No symptoms	11 (23.90)	0 (0)	5 (62.5)	5 (45.45)	1 (20.00)	25.79	0.04
Low somatic symptom severity	10 (21.74)	0 (0)	1 (12.5)	1 (9.09)	2 (40.00)		
Moderate somatic symptom severity	15 (32.61)	1 (50.00)	1 (12.5)	3 (27.27)	0 (0.00)		
Severe somatic symptom severity	10 (21.74)	1 (50.00)	1 (12.5)	2 (18.18)	2 (40.00)		

Symptoms of anxiety and somatization were more frequent in adolescents with myalgia than with other diagnoses, while adolescents with disk displacement with reduction presented greater depression (Table 7). The frequency of symptoms of anxiety, depression and somatization in subjects with myofascial pain, arthralgia and headache was low (Table 4), even when the associations between anxiety (Fisher 25.57, p = 0.04), depression (Fisher 29.23, p = 0.03) and somatization (Fisher 25.79, p = 0.04) and TMD were statistically significant (Table 6). All associations were found in the rural zone (Table 7).

**Table 7:** Prevalence of psychosocial factors in the urban and rural zone. Association with temporomandibular disorders

Variable	Urban zone			Rural zone		
	Prevalence n (%)	Association with TMD Pearson Chi squared	p value	Prevalence n (%)	Association with TMD Pearson Chi squared	p value
Symptoms of anxiety						
Minimal-No anxiety	49 (54.44)	0.52	0.46	30 (33.32)	7.68	0.006
Mild	24 (26.60)			29 (32.20)		
Moderate	10 (11.10)			16 (17.77)		
Severe	7 (7.77)			16 (17.77)		
Symptoms of depression						
Minimal-No depression	41 (45.57)	0.02	0.89	28 (31.11)	12.66	0.0004
Mild	24 (26.66)			20 (22.22)		
Moderate	15 (16.66)			25 (27.77)		
Moderate-severe	6 (6.66)			7 (7.77)		
Severe	4 (4.44)			10 (11.10)		
Symptoms of somatización						
Low somatic symptom severity	27 (30.32)	0.05	0.81	22 (24.41)	4.00	0.04
Moderate somatic symptom severity	17 (18.82)			29 (32.22)		
Severe somatic symptom severity	7 (7.84)			14 (15.55)		

TMD, temporomandibular disorders

The final models for multiple logistic regression were constructed with all the variables included in the multivariate analysis. In the final models, the Nagelkerke coefficient (measurement obtained with the logistic regression to determine the reliability of the variables included in the model to explain the phenomenon (TMD)), presented a value of 0.39 for model 1 and 0.56 for model 2, which showed that 39% and 56% of the differences between groups could be respectively explained by the variables included in the models (p = 0.008 for model 1 and 0.002 for model 2). The Hosmer–Lemershow test assessed goodness-of-fit of the variables of the model and gave a statistical value for the chi-squared of 9.76 with p = 0.34 for model 1 and 8.31 with p = 0.26, suggesting that the variables contained in the models adequately explained the findings of the analysis.

The multiple-variable analysis with logistic regression, showed living in rural zone, symptoms of anxiety, depression and somatization to have statistically significant association (p < 0.05) as associated factors with TMD (OR > 1), and specifically for pain TMD (OR > 2.3) (Table 8).

**Table 8:** Association of psychosocial factors with TMD and pain-related TMD in Colombian Adolescents

Variable	TMD (model 1)			Pain-related TMD (model 2)		
	OR	95% Confidence interval	p value	OR	95% Confidence interval	p value
Living in rural zone	1.83	1.25–2.32	0.04	3.6	2.32–4.2	0.02
Symptoms of anxiety	2.03	1.23–3.21	0.001	3.2	1.18–4.32	0.01
Symptoms of depression	1.23	1.03–2.45	0.04	2.3	1.82–3.24	0.03
Symptoms of somatization	2.28	1.06–4.15	0.03	2.6	1.45–3.8	0.04

TMD, temporomandibular disorders

## Discussion

The objective of this study was to evaluate the association between psychological factors (symptoms of anxiety, depression and somatization) and TMD in adolescents between 12 and 15 years, belonging to urban and rural zones of Antioquia, Colombia. The main findings were: (1) 40% of adolescents presented some type of TMD; (2) Pain-related diagnoses (Myalgia, Miofascial Pain, Referred Miofascial Pain, Arthralgia and Headache attributed to TMD) were more common than intra-articular ones (Disc Displacement with Reduction was the only found); (3) There was no difference between the TMD present in females and males, but symptoms of Anxiety were higher in females than males and somatization was higher in males than in females. All TMD and psychological factors were more prevalent in 13 year-old adolescents; (3) An association was found between psychological factors (symptoms of Anxiety, Depression and Somatization) and TMD in adolescents from rural zones and overall, all the studied psychological factors were associated with TMD.

The frequency of TMD in adolescents, described in studies conducted in different regions of the world, varies between 20 and 46.1% [33–36]. Among these, the studies carried out in South America, have found a frequency of TMD of 46.1% [36], which is slightly higher than that found in this study. As in the available evidence, the results of this research indicate higher prevalence of pain-related disorders than articular disorders [9]. Despite this similarity, there are some differences when comparing the results of this research with those of other studies. Myalgia and Headache attributed to TMD were the most frequent diagnoses in adolescents included in this study (25.5% and 6.11%, respectively). However, systematic reviews and previous cross-sectional studies, have indicated Myofascial Pain as the most prevalent diagnosis [2, 3, 35, 36]. In this investigation, only two cases were found; one with a diagnosis of Myofascial Pain and another with Referred Myofascial pain. In Myofascial Pain, unlike Myalgia or Headache attributed to TMD, the symptomatology is induced from trigger point pressure, it is located in regions (not in specific points) and in many cases refers to other regions [37]. In Myalgia or Headache Attributed to TMD, pain is not referred and occurs at specific points.

Although evaluating heritable conditions of the population, was not an objective of this study, certain genetic characteristics in Americans and Europeans have been identified in the literature as associated with Myofascial Pain [38]. Genetics could be the explanation about why Myofascial Pain is more common in caucasic populations (Europeans and Americans) than in the population studied in this investigation (latin-american adolescents). However, the same genetic conditions are to be studied in Colombia and maybe other latin-american countries to improve the knowledgment on the field.

TMD are highly related to psychological factors [39, 40]. This was confirmed in this study, where OR values showed statistically significant influence of symptoms of anxiety, depression and somatization on TMD and where all TMD diagnoses were found associated with each of the studied psychological factors. The psychological conditions have shown to be different between urban and rural zones, not only in Colombia [41, 42], but also in other regions of the world [43]. The influence of psychological factors on TMD in Colombia, may have different conditions compared to other countries, from a demographic point of view. For instance, the Human Development Index (HDI) [44] in Colombia, shows that rural municipalities are much poorer than those located in the urban zone; not only in economic terms, but also in opportunities. The global HDI in Colombia is 0.74; However, in the rural zone, it is between 0.50 and 0.65 [44], an important difference with other countries in conflict, where the HDI is almost the same for both zones [44].

Situations such as fear, affliction, social disorder, educational inequality, consumption of psychoactive substances or lack of opportunities, are different when rural zones are compared with urban zones in Colombia [45] and could be affecting the HDI. In the case of the rural zone, the family environment, access to basic health services and demographic isolation, among others, can have a negative influence on the mental health of adolescents [3, 15, 20]. Despite this reality, in the authors' knowledge, this is the first study that evaluates the association between psychological factors and TMD in rural and urban zones.

Looking inside each of the psychological factors studied in the present study, there is an evidence on the relationship between depression and somatization and chronic pain related to TMD [46]. This finding has also been reported in adolescents [47]. Another previous study, also observed the presence of depression in 44% and somatization in 74.1% of subjects of all ages with TMD [48]. These values are higher than those found in the present study (39.13% and 32.61%, respectively for depression and somatization). However, when comparing the results of this research with those of studies in adolescents with TMD, the percentages of depression and somatization are lower (10.94% and 10.64% respectively), even when compared with results obtained also in Latin populations [49]. Regarding symptoms of anxiety, the relationship found here with TMD, was previously found by other authors [50, 51]. It was not possible to make this same comparison between rural and urban populations, since no data were found in the literature.

Physical, psychological, social and hormonal changes, typical of adolescence, contribute to increasing vulnerability to psychiatric disorders such as anxiety and depression, and making adolescents more susceptible to muscle and / or joint disorders [47]. In fact, TMD related to pain can be modified with menstrual cycles in women [52]. The median age of females when they have their first period or menarche is around 12 years of age in US [53] and Colombian girls [54]. Hormonal fluctuations have an effect on the intensity of pain [52] and on the psychological conditions of adolescents [55]. In this study, TMD didn´t present statistically significant differences between males and females. However, the design of this study does not allow to establish a cause-effect relationship between menstrual cycle and pain TMD disorders and/or psychological changes. To reach this type of conclusions, it is necessary to carry out future investigations, with cohorts followed in the long term and paired by gender and age; that allow to determine the association of gender with TMD.

Although the progress of TMD was not an objective of this study, it is important to follow up on cohorts to determine the long-term effect of psychological factors on the severity of TMD in Colombia. The reason is the evidence that demonstrates that psychological deterioration increases the severity and persistence of clinical symptoms related to TMD [56], which affects the quality of life [57, 58], and general health [56].

The results of this study will provide a starting point to determine psychological patterns that may be subject to research to prevent and treat TMD in adolescents and prevent future disabilities. The DC/TMD was validated for several diagnoses and achieved in some cases very high levels of sensitivity and specificity, showing good diagnostic accuracy for TMD in adults. The DC/TMD was developed exclusively in adults and still there is no other validated instrument as good as DC/TMD to TMD diagnosis for adolescents. For this reason, it was decided to use the standard of reference instrument, as has been used in other studies, even when being aware of the limitation [59]. Another limitation of this investigation is the type of sample, which was not paired by gender and the cross-section, which does not allow establishing the associations found as cause or effect. Therefore, it would be important to conduct interdisciplinary research that allows comparing the findings and that involves a sufficient sample to achieve generalization of the results in the Colombian population, as well as designs that allow determining the cause-effect relationship that may exist between psychological factors, demographics and TMD.

## Conclusion

This study indicates Myalgia to be the most prevalent TMD in Colombian adolescents. Pain-related TMDs are associated with psychological factors in the adolescent population of rural Colombia. Symptoms of anxiety, depression and somatization were found to be associated to TMD, even when the frequency was not necessarily severe.

## Data Availability

The datasets used and/or analysed during the current study are available from the corresponding author on reasonable request.

## Abbreviations

TMD: Temporomandibular disorders
DC/TMD: Diagnostic Criteria for Temporomandibular Disorders
TMJ: Temporomandibular Joint
ENSAB: Estudio Nacional de Salud Bucal (spanish)
CNHS: Colombian National Health Study
ICD: International Classification of Diseases
CR: Claudia Restrepo
AMO: Ana Maria Ortiz
ACH: Ana Carolina Henao
HDI: Human Development Index
